# Accomplishing an adaptive clinical trial for cancer: Valuation practices and care work across the laboratory and the clinic

**DOI:** 10.1016/j.socscimed.2020.112949

**Published:** 2020-05

**Authors:** Julia Swallow, Anne Kerr, Choon Key Chekar, Sarah Cunningham-Burley

**Affiliations:** a Centre for Biomedicine, Self and Society, Usher Institute, Old Medical School, University of Edinburgh, Edinburgh, Scotland, EH8 9AG, UK; b School of Social and Political Sciences, University of Glasgow, Glasgow, Scotland, G12 8QQ, UK; c Division of Health Research, Faculty of Health & Medicine, Lancaster University, Lancaster, LA1 4YG, UK; d Usher Institute, Old Medical School, University of Edinburgh, Edinburgh, Scotland, EH8 9AG, UK

**Keywords:** UK, Genomic medicine, Clinical trials, Cancer, Valuation practices, Backstage labour, Care

## Abstract

A new generation of adaptive, multi-arm clinical trials has been developed in cancer research including those offering experimental treatments to patients based on the genomic analysis of their cancer. Depending on the molecular changes found in patients’ cancer cells, it is anticipated that targeted and personalised therapies will be made available for those who have reached the end of standard treatment options, potentially extending survival time. Results from these trials are also expected to advance genomic knowledge for patients in the future. Drawing on data from a qualitative study of one such trial in the UK, comprising observations of out-patient clinic appointments, out-patient biopsy procedures, laboratory work, and interviews with practitioners, this paper explores how the clinical and research value of one such trial was accomplished in everyday practice by focussing on the work of clinical trials and laboratory staff across recruitment, laboratory analysis, and results management. In the face of numerous potential set-backs, disappointments and failure, we explore how practitioners worked to balance the need to meet established measures of value such as numbers of patients recruited into the trial, alongside cultivating the value of positive affects for patients by managing their expectations and emotions. This care work was performed primarily by practitioners whose roles have historically been devalued in healthcare practice and yet, as we show, were critical to this process. We conclude by arguing that as complex multi-arm adaptive trials become more commonplace, we need to attend to, and render visible, the dynamic and care-full valuation practices of backstage practitioners through which experimental biomedicine is accomplished, and in doing so show that care both achieves clinical and research value, and is also a series of practices and processes that tends to tissue, patients and staff in the context of ever-present possibility of failure.

## Highlights

• Experimental genomic based trials are difficult to accomplish in practice.
• Valuation practices happen throughout recruitment and trial participation.
• Value was made via coordinative practices of care in the context of failure.
• Staff balance clinical and research value alongside expectations and emotions.
• Vital backstage practices of care were performed by devalued healthcare staff.

## 1 Introduction

Scientific advances in cancer research have led to techniques for understanding the molecular profile of cancer tumours and subsequently the development of targeted therapies and treatments. Randomised control trials, the ‘gold standard’ of evidence-based medicine (see Cartwright, 2007), have been superseded by what Keating and Cambrosio (2011) describe as a ‘new style of practice’ in medical oncology, which is based on large scale, multi-sited trials to develop targeted therapies for subtypes of cancers based on genomic profiling (See also Berry, 2015; Shojaee and Nana-Sinkam, 2017). Umbrella adaptive multi-arm trials are currently being developed and trialled in the UK for some cancer types. An umbrella trial stratifies patients with the same type of cancer to different treatment arms based on the molecular profile of their tumour. A multi-arm trial involves testing several different treatments at once. If a particular drug is not proving efficacious the trial arm can be closed and new treatment arms brought in (see Medical Research Council, 2014; West, 2017). The clinical value of these large-scale, adaptive trials for patients is that, depending on the molecular changes found in patients' cancer cells, targeted and personalised therapies may become available at different points on the treatment pathway and not only when they have reached the end of standard treatment options, potentially extending survival time. The purported research value of these trials is that they will advance genomic knowledge for patients in the future.

Accomplishing these trials alongside routine diagnostic work can, however, be difficult: trial protocols for tissue collection and analysis are complex and continually subject to revision, it is critical that tissue is obtained and analysed in a timely manner, and molecular profiling relies on obtaining tissue of sufficient quantity and quality for analysis, which is difficult given that this often involves invasive needle biopsy procedures (Hiley et al., 2016). These procedures are not without risk for patients and the subsequent samples obtained are not always of the quality or quantity necessary for molecular testing (Hiley et al., 2016). The rate of progression of lung cancer and the fact that patients often present when the cancer is advanced also means that this patient population is often very unwell, thus limiting their eligibility for trial participation. Eligibility criteria for the screening study and subsequent trial include patients who have been diagnosed with Stage 3 or Stage 4 non-small cell lung cancer and with low Performance Status (a measure used to quantify cancer patients' well-being and activities of daily living), which can also be a barrier to recruiting patients.

In this paper, we focus on one such large-scale trial for patients with advanced non-small cell lung cancer that is currently being conducted in the UK. Contributing to Science and Technology Studies (STS) and sociology of medicine literatures on valuation practices and care work in experimental biomedicine, we trace how a complex trial was accomplished in everyday practice by focussing on the work of clinical trials staff and laboratory staff. We explore how clinical and research value was cultivated, investigating the kinds of work that this involved across recruitment, laboratory work, and managing results (including their absence) in the context of potential setbacks and failure. In doing so, we focus on ‘valuing’ as a prominent series of activities (see Dussauge et al., 2015; Heuts and Mol, 2013) in experimental biomedicine; expanding the definition of ‘valuing’, which is predominantly used in the economic sense to denote monetary worth (see Graeber, 2002), to include other benefits of participation valued by patients and professionals, especially positive emotions produced by the work of care-workers such as clinical trials staff (Allen, 2014; Tronto, 1994). The value of the trial was not solely a matter of meeting established metrics of clinical and research value (patient recruitment, retention, sample viability, progression-free survival), but also about successful management of advanced cancer patients' expectations and emotions (Heuts and Mol, 2013).

### 1.1 Promissory rhetorics, valuation practices and care work

The ‘genomic turn’ in cancer clinical trials (Nelson et al., 2014) has primarily been studied by sociologists interested in epistemic and institutional developments in biomedicine and technoscience (see Cambrosio et al., 2017; Keating and Cambrosio, 2011; Kohli-Laven et al., 2011), with only a few studies exploring practitioners' and patients' embodied experiences of trial participation (see for example, Brown and de Graff, 2015). Whilst scholars such as Keating and Cambrosio (2011) have explored the continually shifting organisational and institutional practices and procedures which render large-scale research workable, less attention has been given to the everyday practices involved in trial work (Dussauge et al., 2015; Helgesson and Krafve, 2015; Heuts and Mol, 2013).

Experimental large-scale research such as adaptive multi-arm trials promise more precise, personalised treatments and extended survival for cancer patients both now and in the future. Scholars in the field of the sociology of expectations (Borup et al., 2006; Brown, 2015) argue that promissory rhetorics perform particular values or ‘worths’ cultivating specific ‘matters of concern’ (see Dussauge et al., 2015; Latour, 2004). This is part of the wider bioeconomy of promissory capitalism, where disease-free futures are continually re-envisaged (see Cooper, 2008; Michael, 2000) and where expectations about personal and collective futures feature prominently (see Del Vecchio Good et al., 1990; Haase et al., 2015; Novas, 2006). As Brown and de Graff (2015) demonstrated in their analysis of the lived experiences of advanced cancer patients involved in phase II and phase III randomised control trials, however, both hope and despair are key to trial arrangements in practice (see also Cooper, 2008; Cooper and Waldby, 2014; Will and Moreira, 2010). Negotiating the promissory rhetorics of contemporary biomedicine in practice therefore involves the cultivation of low expectations as well as hope (Gardner et al., 2015).

Contributing to discussions concerning promissory rhetorics and the wider bioeconomy of biomedical innovation, sociology and anthropology literatures have shown the way in which materials (tissue and other objects) at the centre of biomedicine become *conduits* for value as meanings are inscribed in their forms, their uses, their trajectories' (Appadurai, 1986: 5). As Street (2016: 956) argues, precious tissue, scientific, medical and patient labour, and technologies are part of regimes of ‘relational, epistemic and economic value’ (see also Kelly and Geissler, 2013; Rajan, 2006; Waldby and Mitchell, 2006). Vital biological materials like stem cells and embryos have been shown to acquire ‘biovalue’ in the field of regenerative medicine in particular (see for instance, Gardner and Webster, 2017; Hauskeller and Beltrame, 2016; Lee, 2016; Mitchell and Waldby, 2010; Tupasela, 2006; Waldby, 2002). Vermeulen et al. (2011) have also shown how other tissues rendered alien or waste for patients – tumours for example – can be reconstituted as valuable scientific resources in the bioeconomy (see also Street, 2016). Across these literatures, value is primarily framed in economic terms: materials become conduits for value and contribute to the wider bioeconomy of experimental medicine.

In this paper, we extend discussions concerning the value of experimental biomedicine by attending to the everyday practices through which a complex genomic based trial was accomplished. We ask, ‘what comes to count as valuable, desirable, or otherwise worth caring for’ (Dussauge et al., 2015: 10) and who was involved in this work? In doing so, we situate our analysis within the interdisciplinary study of valuation practices, which focuses on the enactment of value through socio-technical assemblages (see Dussauge et al., 2015). ‘Valuing’ is a set of activities and practices to render something ‘good’ or ‘successful’ (Dussauge et al., 2015; Heuts and Mol, 2013). As Heuts and Mol (2013) show in their analysis of what encompasses a ‘good’ tomato, various activities are employed to make tomatoes ‘good’ and yet despite these efforts, ‘success can never be guaranteed’ (p.125). In this paper, we explore how value from this genomic based trial was cultivated amidst numerous challenges associated with its implementation, focussing on ‘sustained and respectful tinkering towards improvement’ (Heuts and Mol, 2013: 125) and on-going care work (Puig de la Bellacasa, 2017).

Following Puig de la Bellacasa (2015), we investigate care as a ‘material vital doing’ and an ‘affective state’ and pay attention to ‘discomfort, unease, and trouble in matters of care’ (Murphy, 2015: 721), including the uncertainties, anxieties and disappointments of participation (Puig de la Bellacasa, 2017: 42). We consider how patients' hopes and expectations were managed alongside the tissue (the gatekeeper to the trial) via ‘care from many different bodies to “realize” what is assumed to be their vital potential’ (Lee, 2016: 460).

In focussing on valuation and care, we render visible the work of clinical trials staff and laboratory practitioners whose roles have historically been devalued in healthcare practice and biomedical research. We trace the embodied and affective practices through which care was performed in the laboratory (see Kerr and Garforth, 2015; Myers, 2008; Star, 2007; Star and Strauss, 1999) and the clinic (see Allen and Hughes, 2017; 2014), including the backstage practices of practitioners acting as ‘intermediaries between the bedside and the laboratory’ (Bourret et al., 2011: 817). Our focus is on how these labours became ‘generative doings’ in the accomplishment of experimental biomedicine and in negotiating the complexities provoked by these large-scale genomic based trials (Puig de la Bellacasa, 2017: 54).

## 2 Methods

This article is based on a qualitative study of a screening study and subsequent umbrella adaptive multi-arm clinical trial in the UK. The trial was multi-sited but the fieldwork we draw on here was conducted in one of the participating institutions as part a wider programme of Wellcome Trust funded research exploring experiences of genomic techniques and tests within oncology research and practice (Translations and Transformations in Patienthood: Cancer in the post-genomics era). The trial we followed was part of a national project aiming to advance treatment for people with lung cancer, funded by a research charity in collaboration with industry partners. The aim of the trial was to determine the benefit of treatments for individual patients based on the genomic profile of their cancer tumour and to assess changes in circulating DNA in the patient's blood to identify drug resistance. The protocol involved a screening study which determined eligibility to be part of the trial. At this stage, patients gave their consent for surplus tissue from routine biopsies to be analysed using next-generation sequencing technology; their results determined their eligibility for entry into the adaptive trial. If patients consented to the screening element of the study, the histopathologists in the histology laboratory prepared the surplus tissue from routine biopsies for DNA extraction in cytogenetic laboratories, which included marking which part of the sample to carry out the extraction. This required histopathologists to negotiate routine diagnostic work with clinical trial work which could be difficult given that diagnostic work takes precedence in these laboratories. The samples were then sent to another institution for analysis and if a full successful genetic panel was returned then patients were asked to consent to be entered into the multi-arm trial. Entry into the trial required patients to consent to a further separate biopsy procedure and the tissue was then prepared in histopathology, DNA extracted in the cytogenetics laboratories, and then further analysed using next generation sequencing at a separate institution to direct entry into one of the trial treatment arms.

Consultant oncologists introduced the screening element of the study to patients and the senior clinical trials assistant was responsible for recruiting patients and seeking consent. If patients were eligible for the multi-arm trial following analysis of the screening study samples, then consultants approached patients about the trial and both the research nurses, and the senior clinical trials assistants, were responsible for recruiting eligible patients to the trial. These practitioners were involved in organising the re-biopsy procedures, the tracking of the samples, and in liaising directly with the laboratories and separate institution. They were also the first point of contact for patients on the treatment arms and were heavily involved in their care throughout their time on the trial.

Fieldwork was conducted over a one-year period between 2017 and 2018 and included 27 observations of specialist multi-disciplinary team (MDT) meetings, out-patient clinic consultations, biopsy procedures, and cytogenetic and histopathology laboratories where the samples were analysed and processed. Seventeen observations of clinical consultations were carried out to explore how patients were recruited to the screening study, the results of which determined eligibility to the trial. We also observed two biopsy procedures and three specialist MDT meetings to explore how eligibility to the trial was negotiated. Five observations were carried out in the cytogenetics laboratories to examine how both the samples from the screening element of the study as well as the samples from the biopsy for entry into the trial were analysed and DNA extracted. Observations were recorded in handwritten notes which were typed up and shared with the project team.

In-depth interviews were also conducted with health care practitioners involved with the screening study and trial, including four consultant oncologists, two pathologists, one clinical trials assistant and two research nurses to gather a wide range of perspectives and experiences of the trial, and to capture the different epistemic cultures which made up the screening and trial team. We were unable to interview the cytogeneticist involved in DNA extraction due to the fact that they moved to a different laboratory at another institution during the fieldwork. One interview was carried out with each practitioner and during the interviews, we focussed on their views on the screening study and trial and its challenges, which included accessing, analysing and processing samples, and discussing the research and results of genomic analysis with patients. Interviews were audio-recorded and transcribed verbatim. We adopted a situational analysis approach to analyse interview transcripts and fieldnotes thematically, dealing with data manually to avoid becoming overwhelmed by quantity and scope (see Clarke et al., 2016). The research project was approved by the relevant NHS Research Ethics Committee [REC number: 16/YH/0229].

The complex design of the adaptive trial was a key challenge for practitioners and included continual negotiation of complicated protocols, efforts to ensure the sampling and analysis of tissue occurred in a timely manner (tissue had to be provided to another institution for analysis in a specific timeframe and in a particular format), and negotiation of the difficulties associated with obtaining the tissue which was not straightforward, especially when it involved needle biopsies. Practitioners' handling of these issues was further complicated by organisational challenges, including staff shortages. The above factors contributed to the institution's low rates of recruitment to this trial in comparison to some other centres, yet there was a prevailing sense of the need to manage these difficulties in order to make the trial a ‘success’.

In what follows, we explore how value was realised by clinical trials assistants, nurses and laboratory staff. We begin by exploring how patients were recruited to the screening study – the first gateway to the trial. We analyse how the clinical trials assistant produced clinical and research value by meeting recruitment metrics at the same time as they cultivated positive affects of participation for patients in the trial by managing, and at times lowering, their expectations. We then go on to capture how practitioners across the laboratory and the clinic worked to contain the precarity of the tissue to maximise participation in the trial. Activities of valuing included caring for the tissue (Lee, 2016) to optimise its clinical and research value, and to minimise disappointments for patients for whom this was their last possible chance for treatment. In the final section, we explore how value to patients was generated despite the ever-present possibility of failure, as the trial created opportunities for staff to care for unwell patients by opening up time and space for patients to display emotions.

Across the paper, we argue that such value was generated by care as a ‘material vital doing’ (Puig de la Bellacasa, 2017) as practitioners acknowledged and (re)configured patients' expectations and emotions in response to the challenges associated with implementing the study and trial (c.f. Heuts and Mol, 2013). Negotiating patients' options and emotions through the study and trial process was key to the work of practitioners, operating alongside their efforts to meet established measures of value in a highly bureaucratised and complex trial. By surfacing these different ways in which the trial was rendered valuable for patients participating, or seeking participation, we show that valuation practices encompass and entwine metrics, tissue and affects.

### 2.1 Practices of recruitment to the screening study: managing expectations

In this section, we explore how patients were recruited to the screening study by drawing predominantly on observations and conversations with the Senior Clinical Trials Assistant (SCTA1) responsible for leading patient recruitment. At this stage of recruitment, patients diagnosed with advanced lung cancer were asked to participate in the study providing they met the eligibility criteria. These patients may have already been on treatments such as chemotherapy or radiotherapy or they may have already had their tumour tested separately from the study and prescribed treatments which target ALK or EGFR amplifications or mutations. The study and subsequent trial provided patients with the possibility of further treatment once the cancer had stopped responding to standard therapies.

SCTA1 was responsible for patient recruitment and worked alongside consultant oncologists to recruit suitable patients to the screening study. Following routine out-patient appointments, the consultant briefly discussed the study with patients thought to be suitable and directed them to SCTA1 for further information. SCTA1 spent approximately 30 min with the patient and family member/accompanying person talking through the study and seeking consent where appropriate. Meeting recruitment targets for the screening study, was a vital part of SCTA1's work. Prior to each clinic the practitioner screened the clinic lists for ‘possibilities’ (Observation 2 Out-patient Clinic) stressing during interview with the researcher the need to ‘keep trying’ despite difficulties associated with recruiting unwell patients. At other times SCTA1 expressed concern that the ‘numbers are really bad’ (Observation 3 Out-patient Clinic) when it was time to submit monthly recruitment figures to the charity funding the screening study and trial. SCTA1 was therefore constantly on the lookout for patients to recruit, which included waiting around in clinic corridors to ‘catch’ patients and following up with, and cajoling, consultants in an effort to meet recruitment targets.

During consultations with patients, SCTA1 also worked to underplay the possibility of the study delivering actionable results. Minimising expectations about the possibility that results from the trial would change treatment decisions in the short term involved framing the results of the genomic analysis as of potential value in the future, focussing on the need to not worry in the present. For example:

SCTA1 asked if the patient had had a biopsy … and then explained that they require a blood sample to send off to [hospital in south of England] which may ‘potentially help’ with future treatment but *not to worry about the results* as it'll be a bit of time before they receive the results from the test.

(Observation 4 Out-patient Clinic)

The complexity of the process and the precious materiality of the tissue were further reasons for keeping patient expectations in check and allowing staff to take on the mantle of worry on their behalf, as illustrated in the extract below:

SCTA1 explained to the patient that ‘it may take a while for the results to come back from the teaching hospital, we may have to run them more than once. We're working with tiny bits of tissue and “perfecting the process”’. The family member nodded. The patient hadn't spoken yet. SCTA1 reassured them by saying ‘don't worry, I'll worry about everything for you’.

(Observation 9 Out-patient Clinic)

Alongside the work of meeting recruitment targets and reducing participants' worries, SCTA1 also sought to ‘bracket’ patients' hope for success to avoid disappointment and anxiety. As SCTA1 described during one conversation after she had consented a patient to the study, ‘I didn't want to get the lady's hopes up’, a point she reiterated during interview when discussing recruitment,

Yeah. I don't give them any hope. I know that sounds really hard actually, I try and just make them realise it's a test and we'll get it back and we'll cross that bridge when we get there.

(Interview SCTA1)

The potential for worry and concern evoked by the study results not coming back quickly or the tests having to be re-run was therefore minimised for patients and families. This required affective labour on the part of practitioners, who described ‘tak[ing] on worry’ and feeling ‘guilty’, as elaborated here:

You feel guilty because you think – if I've put a patient into [the screening study] and I know that their results are not gonna pass, I feel guilty for entering them into the trial… I feel guilty on one side, it's kinda like the good and the evil angel but then I think well, maybe it will pass. But then when it doesn't pass it's like, oh god, just wasted their time and built their hopes up. And I don't wanna do that to patients. I wanna see those results pass but we can't do that unless we bring more and more patients in.

(Interview SCTA1)

Elsewhere SCTA1 also described feeling hopeful about recruitment of new patients as in the extract below:

As the clinical trials assistant walked back into the room following a discussion with the consultant about patient eligibility, she said, ‘the patient may be a good 2 (referring to the patient's performance status), I live in hope’.

(Observation 1 Out-patient Clinic)

Here, we see how SCTA1's work involved carefully balancing the need to meet recruitment targets whilst acknowledging and attending to patients' expectations and potential for disappointments and failures. For other practitioners, this included changing how the study was explained to patients as Consultant Oncologist 4 explained,

We've changed how we've sold [it]. We've sold it as a screens study rather than saying, “You might be eligible for the [trial]” – ‘cos most people won't be.

(Interview Consultant Oncologist 4)

At other times recruitment sat in tension with what practitioners described as patients’ best interests. This included moments where practitioners did *not* recruit, as demonstrated during an observation with SCTA1:

I hate it when people ask you why you haven't consented. I feel like saying, ‘you come to clinic for a day and see what it’s like’, you see the patients crying in the waiting room and with this guy, the fact he can't even walk alone or lift his shoe off the ground. I'm not going to consent just to get the statistics and I will tell people that.

(Observation 8 Out-patient Clinic)

As SCTA1 explained, recruitment to the study and therefore subsequent trial involved more than managing ‘statistics’ to generate potential clinical and research value via recruitment. Valuing here meant actively displacing recruitment in favour of caring for unwell and emotional patients.

Through these activities we see valuing in recruitment work as multi-faceted and dynamic as practitioners sought to meet recruitment targets, encourage sufficiently positive but not excessive expectations amongst patients, and reduce the worry of participation. At other times patients' health and wellbeing were prioritised over recruitment to the trial. Through this work, practitioners cultivated value from the trial, trying to make it a ‘success’ for individual participants and future patients, all the while managing the potential for failure. As we now go on to discuss, these activities were closely connected to deriving value from the tissue, which was used to direct entry into the trial.

### 2.2 Handling tissue across the laboratory and the clinic: containing precarity

Trials depend on tissue being obtained and analysed in a timely way given that tissue can degrade over time and become unsuitable for genetic testing (Hiley et al., 2016). This becomes even more important in trials with very unwell patients who are running out of time and available treatment options.

Participation in the trial we studied was highly dependent on tissue passing quality checks and on successful extraction and sequencing of DNA. To maximise the value of the trial for prospective participants and future patients more generally, practitioners sought to improve the likelihood of successful entry to the trial by caring for the tissue in order to maintain its viability. This happened during laboratory analysis and DNA extraction of the samples for the screening study and multi-arm trial, the biopsy procedures for entry to the multi-arm trial, and subsequent laboratory work to prepare the samples to be sent to a separate institution for further analysis. In these contexts, the tissue emerged ‘not as a waste product, evacuated from the body, nor simply as a biological resource… but as a product of care’ (Lee, 2016: 458), where staff were ‘taking care of [its] potentials and promises anchored to them’ (Lee, 2016:ibid.: 472).

The following extract is taken from an observation with a cytogeneticist carrying out the initial DNA extraction on a patient's tissue sample for the screening element of the study. Provided there was adequate DNA for extraction, the sample would be sent for genomic analysis and the subsequent results could direct involvement in the multi-arm trial. The cytogeneticist described the complexities of sample size and quality in relation to the hopes and expectations for the study:

Before the practitioner began the scraping process, I asked how important it was for the sample to be of good quality (and what this constitutes) before it reaches cytogenetics. The geneticist responded, ‘oh very important but it's difficult as some of the samples are so small’. At this, the geneticist pointed to one of the patient's samples (no bigger than 5mm in diameter) and said ‘you know that's a very small sample that I don't think we'll get a lot of DNA from but we'll try, it's always worth a try. You can just tell by looking at some. What is dispiriting is when it completely fails and this happens a lot with [this study] – we want to do all we can but it's difficult.

… the time frame from consent to when the samples get sent to the other institution … can be difficult for the patient – ‘they may die in this time, turnaround in the other institution can be 21 days. For these patients this is often their last chance’.

(Observation 1 DNA extraction for the screening study in a cytogenetic lab)

Here, the scientist tried to extract DNA to give patients a chance, trying to overcome the difficulties of small samples and minimise delays. Other cytogeneticists noted the urgency of analysis and kept patients in mind through their work. For example, one commented: ‘because it's for a trial I don't like to leave it hanging around in the system’ (Observation 1 DNA extraction for the adaptive trial in a cytogenetic lab) and others spoke about the need to speed up the process, to handle samples in a timely manner because of the precarious position of patients. The tissue subsequently emerged as a proxy patient, to be cared for in the best way possible, and to secure its position as gatekeeper to the trial as it moved between the clinic and the laboratory.

The team associated with collecting and sending the tissue to the laboratory also focused on trying to keep the patient in mind in the laboratory, as Research Nurse 1 explained during interview:

The bit that was left here went to cytology, they couldn't complete the [molecular profiling] test [related to access to targeted therapies] because they didn't have enough of it, it was exhausted, it was insufficient. So we'd sent it off to the trials office for [biomarker] testing [related to immunotherapy], they did the [biomarker] testing, then they did the [molecular profiling] testing as well… I've asked for it, the block to be sent back for [the screening study], but didn't realise that they've actually taken another sixteen slices off it, so there's a little tiny bit left, so we've just asked [name of pathologist] to see if there's enough in there to send off for [the screening study]. The reason I've done this is because I know this patient's started to progress. So I've got to be thinking all the time, well what's the next step…

(Interview Research Nurse 1)

In this account, the nurse sought to keep track of and stave off the deterioration of patients and of tissue, keeping a sense of what was possible and how tissue and patients changed over time; balancing competing priorities and opportunities. This involved negotiations with oncologists, other nurses, pathologists and scientists to try to maximise the quality of the tissue to ensure it did not become ‘exhausted’ by routine diagnostic work as well as other kinds of analysis outside of this study/trial. This was especially important for patients who had deteriorated and were no longer responding to standard treatment: practitioners had to capture the tumour in the right ‘state’ of mutation, somewhere between the disease progressing too little (when responding to treatment) and too much (when the patient becomes too unwell to proceed with another biopsy), described by Consultant Oncologist 7 as ‘sequential management’.

We also found that nurses were particularly involved in maintaining or valuing patients’ interests via efforts to ensure appropriate and timely preparation and analysis of tissue in histology labs, as SCTA1 described:

It takes absolutely forever for a sample to get cut… the histology departments don't see it as an urgent type of thing. There needs to be a lot of education going into histology and why we are asking for these samples to be cut, and what those samples actually mean to that patient. That is huge because I think sometimes they just end up at the bottom of the pile and… they just add more and more and more on top … And why do I have to prod them [scientists] to do it? I shouldn't have to do any of that.

(Interview SCTA1)

In this excerpt, SCTA1 frames her role as the guardian of patients' interests via the careful stewardship of tissue, freighting it with meaning and urgency for patients and worrying that this may not be a priority in the histology laboratory where diagnostic work takes precedence. Her concern was to remain attentive to and negotiate the competing priorities of histology laboratories and cytogenetic laboratories. This meant that SCTA1 was sometimes present in the biopsy procedure for the multi-arm trial to ‘pass over’ tissue to the histology lab and keep close to the patient to ensure the tissue did not get ‘lost’ as below:

As we left the consultation room the practitioner handed me the sample explaining that she needed to message the pathologist leading the trial to inform them it would be heading to the lab for analysis – ‘covering my back after the last sample was lost’. On arrival at the lab, SCTA1 whispered to me as we waited for the pathologist to arrive, ‘this is where it all goes wrong.’ I responded and asked if she worries about what will happen to the sample and she explained that she feels responsible as she doesn't trust that it will reach the right person or be analysed in time for the DNA quality to remain intact… ‘got to hope it's handled in a timely way’.

(Observation 1 Adaptive Multi-arm Trial Biopsy)

In this discussion, there is a sense of the patient as an absent yet vital party, and the practitioner as an advocate on their behalf. Other similar kinds of ad hoc work to ensure the tissue was ready for analysis included nurses prompting doctors to write onto vials and forms where vials were marked as ‘urgent’ for analysis – ‘this wouldn't harm the process if it is fast tracked’ (Observation 2 Adaptive Trial Biopsy). The diagnostic work in histology laboratories however took precedence over clinical trial work, requiring research nurses and clinical trials staff to work closely with staff in these labs to emphasise the importance of timely analysis, minimise the likelihood of failure, and draw their attention to the patient waiting on the results.

These multiple activities of valuing involved caring for the tissue alongside managing patients' expectations: negotiating competing priorities and opportunities to improve patients’ health and wellbeing, including choreographing re-biopsy procedures for possible entry into the trial. Tinkering towards improvement and cultivating the possibility of success involved valuation practices across laboratory and clinical settings where the possibility of failure was ever-present.

In the final section, we look more closely at how the prospects and realities of failure were managed, including by the cultivation of other kinds of value for patients and practitioners. We focus in particular on practitioners' framing of participation as a valuable opportunity to do emotional work to support patients and manage their own feelings of guilt and disappointment. These kinds of ‘material vital doing(s)’ (Puig de la Bellacasa, 2017) were backstage but a key aspect of the valuation practices of the trial.

### 2.3 Managing results and their absence

Feminist approaches to care demonstrate how this kind of work is often devalued as ‘unproductive’ (Adam, 2004: 127) but remains vital to social life. As we show in the following section, caring for patients did not always generate value in the form of recruitment to trials or effective treatments, but it was critical to making the prospects or realities of potential failure more bearable by generating other kinds of positive affects for practitioners and patients.

For those patients who ‘pin their hopes on the test’ (Observation 4 Lung cancer clinic), as a way to access novel experimental treatments, practitioners carefully crafted both time and space to respond to their concerns, potential anxieties and disappointments, and in so doing attended to the work of ‘discomfort, unease, and trouble in matters of care’ (Murphy, 2015: 721). The following extract from an interview with Research Nurse 1 highlights the lengths to which practitioners could go to try to get patients onto an arm of the adaptive trial.

We've recently had a patient who has been waiting and waiting and waiting and to be fair the [other institution] have been really, really amazing and they've tried everything to get a result; not to manipulate the result but to try and get a genetic result for this patient, and unfortunately the patient didn't have enough deletions in the gene. They only had one deletion, they needed two deletions, so ultimately the patient wasn't eligible to go into that arm of trial and it's just absolutely heartrending really sometimes, because you know that these patients are at the end of a very long journey and this was the only beacon of hope for them, and they come back as not compatible to an arm of the trial. So it is very hard.

(Interview Research Nurse 1)

The research nurse outlined the work involved in trying to make the trial a success for the patient across institutional settings, holding on to the hope of success, however limited, in the context of failure. This prevailing sense of hope was attached to what SCTA1 described as searching for the ‘unicorns’ who would respond well to treatment,

And that I think is kind of what keeps us going, is that maybe there could be more unicorns.

(Interview SCTA1)

When faced with patients being unable to enter the trial, practitioners also spoke of the importance of carrying out the emotional work of trying to make the best out of failure by making time for patients and listening to their concerns, as SCTA1 explained,

I just let them talk, yeah… I just let them talk about what – whatever they want really and just listen … most of the time they just really want somebody to listen to them.

(Interview SCTA1)

However, carefully navigating potential failure meant practitioners navigating disappointments and their own ‘guilt’ at being unable to deliver a positive result for entry into the trial, which at times meant caring from a distance as discussed below:

Research Nurse 1 told me about a patient who keeps ringing at least twice a day to ask where their results are – she keeps having to tell the patient that they don't have results to feedback as the test has failed…we discussed the fact that this makes conversations with patients very hard…Research nurse 1 then told SCTA1 about the patient who keeps ringing ‘I don't know what to do’ (SCTA1). At this point SCTA1 quickly scans the appointment sheet in front of them and realises that this lady is in clinic. The trials assistant remarks on how ‘guilty’ she feels not being able to give a positive result (no result despite re-biopsying) and that if she came across the patient again, she ‘wouldn't know what to say’; ‘it's hard for me, never mind the patient’. SCTA1 describes the effort she goes to, to avoid the lady in clinic including walking around the perimeter of the clinic rather than through the waiting room which is exactly what we did when we left.

(Observation 1 Out-patient Clinic)

Whilst avoidance has the potential to be seen as ‘careless’ (Mol et al., 2010) we argue that the avoidance described above is in fact emblematic of practitioners' work to protect and care for patients, which at times meant not being in touch. Practitioners had to carefully navigate when or if to engage with patient concerns to protect both themselves and patients from negative emotions. In so doing, they maintained a sense of the trial being valuable *despite* delays and anxieties. Research nurses were key to this work, as discussed during an interview with Consultant Oncologist 7:

It's hard for everybody.And as you say, probably me as a clinician will get less.They'll listen to me but then they'll agonise over it and ring the research nurses and say, “Well, what happened?” because they feel that they can do that with the research nurses… research nurses spend a long time with them.

(Interview Consultant Oncologist 7)

As this practitioner described, this kind of care offered reassurance and a sense of being cared about for patients and relatives even if they were not able to participate in the trial. As Consultant Oncologist 7 and SCTA1 explained, coordinating time and listening to patients during, before, and after appointments where results or participation were not promising, was another valuation practice which was revealed as critical to the accomplishment of the trial.

Across these caring activities, we can see efforts to derive value for patients from their engagement with the trial, even when they were not able to proceed onto an adaptive trial arm. This included engagement with patients' desire for progress in and beyond clinic appointments, listening to patients' concerns and anxieties, as well as practitioners’ management of their own emotions of frustration and disappointment. It also involved maintaining a sense of the value of the trial by at times avoiding engaging with the anxieties of patients. What was at stake (Dussauge et al., 2015) shifted as attending to affect and emotion was at times privileged and other times superseded by the epistemic and organisational workings of a bureaucratic large-scale research endeavour.

## 3 Discussion

The purpose of this paper has been to investigate how the value of an experimental genomic based trial was cultivated in conditions of complexity and ever-present possibility of failure. Whilst there is an increasing body of literature exploring the institutional and organisational procedures through which large-scale genomic based research is accomplished, less attention has been given to the everyday practices involved in trial work, particularly the backstage practices of those involved in recruitment and laboratory work. In addressing this gap, we have begun to consider how achieving value by meeting recruitment targets or rates of sample viability sat alongside the cultivation of other kinds of value to patients and participants that were not quantified and thereby formally recognised as important to making a success of these kinds of trials.

We have captured how efforts to meet established measures of clinical and research value sat alongside other kinds of efforts to generate positive affects from the trial for patients by managing their expectations and emotions in the context of setbacks and ever-present possibility of failure. We have shown how these valuation practices could coalesce but also work in tension. They involved emotions, bureaucratic processes and lively and precarious tissue, encompassing care as both a ‘material vital doing’ and an ‘affective state’ (Puig de la Bellacasa, 2017). Another key theme in our analysis of these valuation practices are their dynamic nature, encompassing processes of tinkering towards improvement when success could not be guaranteed (Heuts and Mol, 2013). We have also shown that much of this work was ordinary but nevertheless vital. It involved nurses speaking to patients on the phone between appointments, following up on the location of samples and progress of analysis, corridor talk with fellow professionals expressing frustrations and concerns, and avoidance. Laboratory practitioners were also involved in the work of looking after the tissue and trying to achieve trial entry on their behalf.

In tracing such activities of valuing, we have situated our analysis within wider STS and sociology of medicine literatures on value practices and care work, mapping the trial from initial recruitment via the screening study, to DNA extraction and analysis of tissue, to management of results (and their absence). Across the paper, we showed the coordinative practices through which value was cultivated and in doing so extended definitions of ‘valuing’ beyond notions of economic worth, which dominate literatures on experimental biomedicine (Helgesson and Krafve, 2015).

In the first section of the analysis, we demonstrated the work involved in recruiting patients to the screening element of the study where practitioners downplayed the idea that the study and trial would be immediately beneficial for patients during the consent process: anticipating future failure. Minimising or (re)calibrating patients' expectations in an effort to avoid disappointment cultivated positive affects amongst patients, sometimes working alongside efforts to increase recruitment figures whilst at other times superseding such efforts. In the second section of the analysis, we demonstrated the way in which research nurses, clinical trials assistants and cytogeneticists took care of the precarious and yet precious tissue, which was key actant or gatekeeper for entry into the trial. Generating and maintaining the value of the tissue included a variety of backstage practices on the part of these practitioners which at times included stewarding, labelling, and protecting tissue from exhaustion as it moved between different tests, departments and institutions in an effort to contain its precarity and maximise success. Practitioners worked to cultivate the tissue as vital material and in doing so secure its clinical and research value. We suggested that the tissue also emerged as a kind of ‘proxy-patient’ (cf. Parry, 2018) to be cared for in order to cultivate clinical and research value as well as patients' emotions, offsetting and minimising potential disappointments and setbacks for patients waiting on the possibility of treatment. In the third section of analysis, we drew further attention to the work of attending to affects and emotions when entry to the trial was foreclosed. We focused in particular on efforts to manage the absence of positive laboratory results, exploring how practitioners dealt with uncertainties and anxieties when success could not be guaranteed, variously trying to maintain positivity, a sense of the value of the trial, and to offer comfort to some patients, whilst caring at a distance for others (Heuts and Mol, 2013).

Across the paper, we have shown that generating and cultivating value was more than a matter of the overall success of recruitment and patient entry into the trial, but of cultivating positive affects amongst patients and at times practitioners through sometimes quite ordinary kinds of care work. As Lopez Gil (2007) argues, care as a series of material and affective tasks often makes them difficult to value but, as we demonstrated in this paper, the work of maintaining, repairing or tinkering was critical to making the trial bearable for patients in the advanced stages of cancer who were facing numerous setbacks.

As multi-arm adaptive trials become more commonplace, we need to attend to the dynamic and care-full valuation practices of backstage practitioners which are critical to accomplishing experimental biomedicine and accounting for the ever-present possibilities of failure. In rendering this work visible, we have begun to unsettle and disrupt ‘sedimented arrangements of valuation and devaluation’ (Murphy, 2015: 722) to capture how care is both a way of achieving clinical and research value, and a series of practices and processes that tends to tissues, patients and staff which come to be valued in their own right.

## Author contribution

All authors were involved in conceiving, researching and editing this paper and Swallow and Kerr wrote the paper.
